# Informative value of referral letters from general practice for child and adolescent mental healthcare

**DOI:** 10.1007/s00787-021-01859-7

**Published:** 2021-08-21

**Authors:** S. Aydin, M. R. Crone, B. M. Siebelink, M. E. Numans, R. R. J. M. Vermeiren, P. M. Westenberg

**Affiliations:** 1 Department of Developmental and Educational Psychology, Leiden University, Wassenaarseweg 52, 2333 AK Leiden, The Netherlands; 2 Department of Public Health and Primary Care, Leiden University Medical Centre, Leiden, The Netherlands; 3 Department of Child and Adolescent Psychiatry, LUMC Curium, Leiden University Medical Centre, Oegstgeest, The Netherlands; 4 Youz, Parnassia Group, Rotterdam, The Netherlands

**Keywords:** Referral letter, Child and adolescent mental healthcare, Psychiatry general practice, Diagnostic agreement

## Abstract

Although referral letters (RLs) form a nodal point in a patient’s care journey, little is known about their informative value in child and adolescent mental healthcare. To determine the informative value of RLs to child and adolescent psychiatry, we conducted a chart review in medical records of minors registered at specialized mental healthcare between January 2015 and December 2017 (The Netherlands). Symptoms indicated in RLs originating from general practice (*N* = 723) were coded and cross-tabulated with the best estimate clinical classifications made in psychiatry. Results revealed that over half of the minors in the sample were classified in concordance with at least one reason for referral. We found fair to excellent discriminative ability for indications made in RLs concerning the most common psychiatric classifications (95% CI AUC: 60.9–70.6 for anxiety disorders to 90.5–100.0 for eating disorders). Logistic regression analyses suggested no statistically significant effects of gender, age, severity or mental healthcare history, with the exception of age and attention deficit hyperactivity disorders (ADHD), as RLs better predicted ADHD with increasing age (OR = 1.14, 95% CI 1.03–1.27). Contextual problems, such as difficulties studying, problems with parents or being bullied were indicated frequently and associated with classifications in various disorder groups. To conclude, general practitioners’ RLs showed informative value, contrary to common beliefs. Replication studies are needed to reliably incorporate RLs into the diagnostic work-up.

**Supplementary Information:**

The online version contains supplementary material available at 10.1007/s00787-021-01859-7.

## Introduction

Children’s mental health is an acknowledged key area of concern for overall health, as is the adequate and appropriate allocation of resources available for mental healthcare [1–5]. In many countries the general practitioner (GP) is at the heart of this challenge with its key role in the recognition and referral of those in need of specialized care [6]. The bridge to specialized healthcare is formed mostly by referral letters (RLs). In fact, the RL represents the only substantive information transfer and the starting point for decision making by the receiving services in a considerable number of cases. Evidently, RLs are central to a patient’s transition and can potentially contribute to the diagnostic work-up and subsequent adequate provision of healthcare [7–13]. Notwithstanding, it is a widespread assumption that RLs hold very limited or no substantive value and are merely an administrative task [5].

Several studies across various fields of medicine have analysed the information content of RLs, but little is known concerning the average RL for children and adolescents accessing mental health services [14, 15]. RLs to psychiatric services could potentially guide institutions as regards the urgency of registration or even which subspecialty may be appropriate (e.g., emotional disorders). Studies concerning the recognition of psychosocial problems show variation depending on the type of disorder, generally with lower recognition rates for emotional disorders compared to externalizing or developmental disorders. Within emotional disorders, anxiety disorders are often less well recognized than depressive disorders [16–21]. These variations may well hold when considering the informative value of RLs. Nonetheless, as per our knowledge no study has provided a comprehensive overview of the full range of common reasons for referral, or has addressed the question of the informative value of RLs for child and adolescent mental healthcare.

## Objectives

To increase understanding of the informative value of RLs, in this study we compared information found in children’s and adolescents’ RLs to the later diagnostic classifications made in specialized mental healthcare. First, we asked if RLs demanding urgency were associated with higher levels of functional impairment. Next, we inspected predictive values for the full breadth of diagnostic categories covering higher order level emotional and developmental disorders, and specifically for the common disorder groups: anxiety disorders, depressive disorders, post-traumatic stress disorders (PTSD), eating disorders, autism spectrum disorders (ASD), attention deficit (hyperactivity) disorders (ADHD), and behavioural disorders. In an explorative approach, we also inspected cross-relations between these categories and indications made in RLs. Thirdly, we aimed to relate the predictive value of RLs to age, gender, levels of functional impairment, and length of psychiatric treatment history. In addition, finally, to gain broader insight into the reasons for referral, we examined the informative value of more general reasons for referral mentioned in RLs [5], such as physical ailments or educational and parental difficulties.

## Methods

### Study design and sample

We conducted a retrospective chart review of the electronic medical records (EMRs) at Curium-LUMC, a clinic for mental health treatment affiliated to Leiden University Medical Centre (LUMC). Curium-LUMC receives referrals from a quarter of all municipalities in The Netherlands, and offers outpatient, day patient, and inpatient treatment for minors aged 3–18 years. Outcomes were based on institutional protocols designed to classify DSM-5 diagnoses following the gold standard assessment procedure in child and adolescent psychiatry. The diagnostic work-up facilitates combining structured information from various informants (youth themselves, caregivers and/or teachers), as well as the clinicians’ judgement after interview and observation [16, 22–25].

For the purposes of feasibility we set a 2-year limit and included cases that registered between January, 2015 and December, 2017. To improve the reliability of the reference standard [22] we only included data on cases classified using a comprehensive assessment including interview with a clinician, observation, and a structured multi-informant assessment. The latter was provided by the Development and Well-Being Assessment (DAWBA [26]) which is part of the institution’s intake protocol. Yearly about 30% of the total caseload of the institution follows a different intake and assessment procedure. Those are patients that register for inpatient care or in a critical situation, and are not included in this study. Within the set time period 1268 patients and/or caregivers had completed the comprehensive intake procedure. Three cases were excluded because of an illegible RL, and six owing to the absence of an RL in the EMR. This resulted in a sample of 1259 extracted RLs, of which the 723 (57.4%) from general practice could be included in the study. As this is the first study to investigate RLs for a wide a range of reasons of referral, we decided a priori to analyze only RLs from the most frequent referrer. In The Netherlands, as in many other European countries, this is the general practitioner [27, 28]. An overview of referrers can be found in the supplementary material.

### Data and measures

We coded and then compared which of the various mental health disorders were indicated in RLs and whether they corresponded to the final clinical classifications including comorbidities. Coding followed the DSM-5 chapter structure, e.g., post-traumatic stress disorder (PTSD) and obsessive compulsive disorders were separated from anxiety disorders, whereas phobias were included [29]. For common disorders in psychiatric services, such as ASD and ADHD, we present values for individual classifications rather than a whole chapter (e.g., the neurodevelopmental disorders) combined. Regarding the higher order disorder groups, we present metrics for both internalizing disorders and developmental disorders, rather than the common dichotomisation of internalizing versus externalizing problems. This approach was based on the high prevalence of ASD and ADHD and the very low prevalence of conduct disorders in the study sample, as well as the fact that ADHD is conceptually related to both externalizing and neurodevelopmental disorders. All data were handled in compliance with regulatory requirements and the code of conduct for research using health data. Based on the retrospective nature of the study, the Medical Ethics Committee of the LUMC provided an exemption for written informed consent (G18.080).

### Extraction of referral letter data

RLs were extracted from individual EMRs. Two graduate students transcribed the clinical texts from RLs into a digital data extraction form. To achieve consistency in data extraction, the students and author SA independently extracted an initial set of 30 RLs. After achieving consensus, for each 100th transcribed RL, five selections were examined and discussed to prevent variation developing over time.

An EMR login code that only gave access to filed correspondence was created to ensure blinding for diagnoses recorded elsewhere in a patient’s EMR. The data extraction form included the following: a transcription of the main reason for referral, other contextual information relayed with the RL, whether an ICPC code (International Classification of Primary Care code [30–32]) was included, which ICPC codes were present (together with the year and textual description of the code), the referring healthcare institution, and whether the data extraction should be discussed. The form also captured an approximate summary of the patient’s psychiatric treatment history (no other previous mental health treatments, short-term treatment of up to a year including primary healthcare, or a relatively long treatment history). This is an estimation for whether patients were diagnosed earlier, as an approximation for whether the referrer might have used a formal diagnosis in the RL. To better estimate treatment history, RLs were not our only source to estimate treatment history. Where necessary, students were asked to search for additional information in other correspondence present in the EMR. If RLs were sent and filed with attached reports from earlier treatments, these attachments were not extracted.

### Coding of the referral letters

Regarding indications of urgency, we distinguished three groups of RLs: those in which priority was explicitly requested (including the words “urgent” or “emergency”), in which a serious need was indicated explicitly (“ASAP”, “major” or “serious” [problems]), and those without any such statement.

With respect to reasons for referral, we labelled the transcribed RLs using codes from the ICPC-01 classification system currently used in general practice in The Netherlands [32]. The ICPC system provides codes for reported symptoms and contextual problems, in addition to codes for physician’s (tentative) diagnoses. To aid the coding process, an extensive manual including a glossary of probable reasons for referral and corresponding ICPC codes was compiled and discussed with a GP who has extensive experience with mental healthcare and research using the ICPC coding system. Besides codes from chapter P (for psychological problems), the manual also included codes from chapter Z (for psychosocial problems), as well as some general codes for physical ailments (e.g., A04-Weakness/tiredness, N01-Headache, D01-Abdominal pain/cramps). This manual was refined over the course of five meetings based on the discussion of 20 RLs that were individually coded by SA, PMW, BMS and MRC. During this iterative process some extra codes that are not covered by the ICPC system were added due to their high prevalence in RLs (e.g., self-harm, being bullied, school attendance problems). Based on the length and information load of the RLs, we labelled each RL with up to five ICPC codes and coded in order of decreasing importance (from the main reason for referral to more peripheral symptoms and problems mentioned in RLs).

To evaluate consistency in coding, a random selection of 150 RLs was made and the weighted average agreement was computed between the first author who coded all RLs and the three second coders who each coded a set of 50 letters. Weighted average agreement between coder 1 and the three 2nd coders was 82% (lowest 79%, highest 83%), suggesting generally reliable coding. Chance-corrected agreement on the frequency of specific reasons for referral was also high, for example, excellent agreement was reached on whether anxiety was coded or not, with an overall $\kappa$  = 0.81 (95% CI $\kappa$  0.73–0.86, Online Supplementary Material).

### The reference standard and clinical context

The diagnostic process starts immediately upon registration of a patient and receipt of an RL. RLs are scanned and filed in EMRs. A designated employee then conducts a short telephone interview with parents or caregivers, and provides them with an admission package that includes a login code for the online multi-informant DAWBA tool [26]. Parents, teachers and youth over the age of 11 years are invited to respond, except in case of an inpatient referral. In the online DAWBA environment informants’ responses to closed-ended questions generate scale scores which, together with their responses to open-ended questions, can be remotely reviewed by a clinical rater. A report on this review is then copied to the EMR to facilitate reliability during a face-to-face intake interview that is often led by a senior psychologist. Therein the professional is free in how to incorporate the DAWBA data or to supplement with additional assessment methods. The intake assessment is followed by a psychiatric assessment, after which a classification and a CGAS score [33] is entered in the EMR. CGAS (Children's Global Assessment Scale) scores are an estimation of the level of functional impairment and range between zero and 100, with lower scores indicating more impairment. Depending on complexity and needs, variations to this protocol are common in daily clinical care. The administration of a classification can be postponed when further assessment is needed or the endorsement of a DAWBA is passed when a case enters with emergence. In addition, classifications can be adjusted following insights obtained during treatment. We found, in line with the available literature [34], that such adjustments in classifications were made in about a tenth of cases, over half of which considering minor changes (for example a deletion of a V-code: other conditions). In these instances the last entry was kept as reference. Contrary to the reasons for referral, outcome measures could be extracted groupwise and concurrently from the EMR system [35].

### Secondary measures

To better understand sample characteristics, we obtained data on a patient’s age and gender, their neighbourhood socioeconomic status (nSES) score and the type of care (outpatient, daycare or inpatient). Age and gender were extracted from the DAWBA data, whereas nSES and type of care were derived from the EMR. nSES is a normalized and standardized score based on the income, education and occupation of inhabitants for each postal code area in The Netherlands [36].

### Statistical analysis

First, the demographics of sample and excluded cases were compared in an ANOVA, with nSES and CGAS scores as dependent variables, and sample and type of care as main and interaction effect. This was followed by an analysis of descriptive statistics to gain insight into the content of the average RL.

Using ANOVA, we compared impairment levels (as approximated by CGAS scores) between the three types of referral letters (priority requested, serious problems indicated or normal referral).

The reasons for referral and the final clinical diagnoses were then cross-tabulated for the various classifications. We noted the number of RLs that accurately predicted outcome as a ratio of the total frequency of a psychiatric outcome. This represents the sensitivity of a test and when plotted against the specificity of an instrument the area under the receiver operating curve (AUROC) value is obtained. AUROC values are considered to be insensitive to sample prevalence and indicate the strength of discriminative ability, being graded as fair (0.50–0.70), fair to moderate (0.70–0.80), good (0.80–0.90) and excellent (0.90–1.00) [37]. Plots were created for those with and without multiple classifications to obtain values representative for the daily clinical cohort (including those with comorbidity) and to provide insight into the potential effects of comorbidity on the metrics. AUROCs were plotted using pROC [38] and 95% CIs of the diagnostic metrics were computed in EpiR [39].

We computed positive predictive values (PPVs), negative predictive values (NPV) and likelihood ratios of positive and negative RLs (LR^+^ and LR^−^) to quantify the likelihood of classifications being made. PPVs are computed as the number of RLs classified with their reason for referral as a ratio of the total frequency of that reason for referral. Similarly, NPVs represent the percentage of those who were not referred for a particular problem and were not classified as such, expressed as a ratio of the number of RLs without that particular reason for referral. As a percentage, predictive values are very intuitive. Nonetheless, they depend on the prevalence of the outcome and are, therefore, not easily generalizable. LR^+^ and LR^−^ values, on the other hand, are less susceptible to sample distribution [40] as they represent the actual likelihood of a particular outcome for those positive (LR^+^) or negative (LR^−^) on a test. For LR^+^, values > 2 indicate a slight increase in post-test probability of about 15% in the likelihood of a positive outcome, and > 10 indicates a large increase of approximately 45%. LR^−^ values < 0.5 point towards a slight decrease of 15%, and < 0.1 a decrease of 45%, interpreted as a strong indicator of absence. Tests with an LR^+^ > 20 or LR^−^ < 0.05 are deemed diagnostic in clinical practice [41].

Finally, in a logistic regression analysis, we analysed whether the predictive value of RLs differed depending on age, gender, CGAS score or treatment history.

## Results

### Sample characteristics

Demographics of the sample are depicted in Table 1. On average, girls (43.6%) were 13 years and boys were 10 years. Around a third of the sample had no history of previous mental health treatment. The majority had one (47.4%) or two (27.9%) DSM-5 classifications (Table 2). The study sample had an average nSES score (*M* = 0.47, SD = 0.77) and moderate to serious dysfunctioning as approximated by CGAS scores (*M* = 51.01, SD = 7.61, *n* = 689). The included study sample was similar to the not included caseload of the institution regarding nSES score (*F*_(2, 2032)_ = 0.58, *p* = 0.56, $\eta_{{{\text{partial}}}}^{2}$ < 0.000), but showed a higher CGAS score (*F*_(2, 1804)_ = 14.53, *p* < 0.000, $\eta_{{{\text{partial}}}}^{2}$  = 0.016).

**Table 1:** Sample characteristics *N* = 723

	*n* (%)
Age	
5–7	131 (18.1)
8–10	189 (26.1)
11–13	153 (21.2)
14–15	147 (20.3)
16–18	103 (14.2)
Gender	
Male	408 (56.4)
Female	315 (43.6)
Mental health treatment history	
None	202 (27.9)
Short/Limited	228 (31.5)
Long	284 (39.3)
Unknown	9 (1.2)
Medical conditions	
None classified	577 (79.8)
Singular	47 (6,5)
Complex	18 (2.5)

The Mental health treatment history variable is an estimation based on the information available in the medical record, see below section “data extraction”

**Table 2:** Prevalence of the various clinical classifications

	*n* (%)
Clinical classifications	
Neurodevelopmental disorders	425 (58.8)
Intellectual disability	21 (2.90)
Communication disorder	18 (2.49)
Motor disorders	14 (1.94)
Autism spectrum disorder	214 (29.60)
Attention deficit hyperactivity disorder	243 (33.61)
Specific learning disorder	38 (5.26)
Other Neurodevelopmental Disorders	25 (3.46)
Schizophrenia spectrum and other psychotic disorders	2 (0.28)
Depressive disorders	92 (12.72)
Anxiety disorders	105 (14.5)
Separation anxiety disorder	8 (1.11)
Specific phobia	6 (0.83)
Social anxiety disorder	16 (2.21)
Panic disorder	8 (1.11)
Agoraphobia	1 (0.14)
Generalized anxiety disorder	47 (6.50)
Anxiety disorder not otherwise specified	28 (3.87)
Obsessive–compulsive and related disorders	8 (1.11)
Trauma and stressor-related disorders	39 (5.4)
Post-traumatic stress disorder	21 (2.90)
Adjustment disorder	4 (0.55)
Reactive attachment disorder	15 (2.10)
Disinhibited social engagement disorder	1 (0.14)
Disorder of infancy, childhood, or adolescence not otherwise specified	24 (3.32)
Somatic symptom and related disorders	17 (2.35)
Feeding and eating disorders	27 (3.73)
Elimination disorders	8 (1.11)
Gender dysphoria	6 (0.83)
Disruptive, impulse-control, and conduct disorders	43 (5.95)
Oppositional defiant disorder	15 (2.10)
Intermittent explosive disorder	2 (0.28)
Conduct disorder	2 (0.28)
Other specified- or Unspecified Disruptive, impulse-control, and conduct disorders	24 (3.32)
Substance-related and addictive disorders	2 (0.28)
Personality disorders	34 (4.70)
Number of clinical classifications	
0	91 (12.6)
1	343 (47.4)
2	202 (27.9)
3	71 (9.8)
4	15 (2.1)
5	1 (0.1)

The distribution of the clinical classifications is depicted as per the DSM-5 chapters, excluding the classified V-codes

*NOS* not otherwise specified

There were no cases classified with Bipolar and related disorders, Mutism, Body dysmorphic disorder, Dissociative disorders, Acute stress disorder or Sleep–wake disorders. Cases could be classified with more than one diagnosis

### Content of referral letters

The average extracted reason for referral consisted of 59 words (SD = 41, range 2–246) and depicted problems regarding psychological functioning as well as contextual information. Priority was requested in 36 RLs (5.0%), and a serious need was explicitly indicated in another 50 RLs (6.9%). A few RLs stated only a general request for psychiatric evaluation or treatment without any other additional information (1.2%, Table 3). Most RLs contained one (25.0%), two (32.2%) or three (24.8%) symptoms or tentative diagnoses. The majority of reasons for referral concerned psychological problems. Next to the textual description of the problems which we coded ourselves, 45.8% (*n* = 331) of RLs contained an ICPC code registered by the referrer, most of which were from the P chapter (online supplementary table).

**Table 3:** Frequencies of problem areas in referral letters

	First	Second	Third	Fourth	Fifth
Psychological	685 (94.7)	402 (55.6)	196 (27.1)	82 (11.3)	29 (4.0)
Social	26 (3.6)	113 (15.6)	95 (13.1)	30 (4.1)	12 (1.7)
Physical	3 (0.4)	18 (2.5)	10 (13.8)	9 (1.2)	3 (0.4)
No code labelled at this spot	9 (1.2)	190 (26.3)	422 (58.4)	602 (83.3)	679 (93.9)

Depicted are the frequencies (%) of the coded ICPC codes, per domain, per coding spot (*N* = 723). Psychological = codes from the P and T chapters (eating disorders and symptoms) combined. Social = Z chapter. Physical = all other labels given. On some of the RLs referrers had written ICPC codes themselves—these can be found in the supplementary material

### Informative value of referral letters

The average CGAS score of youth with an RL not explicitly indicating urgency or a severe status (*M* = 51.35, SD = 7.12) was only slightly higher when compared to those with an RL that explicitly mentioned urgency (*M* = 47.27, SD = 8.12) or an RL stating the seriousness of the condition (*M* = 48.83, SD = 8.01; *F*(2,686) = 7.71, *p* < 0.001).

Whereas 41.6% of RLs did not mention any of the later clinically established classifications, the majority of RLs (50.8%) mentioned one, two (7.3%) or even three (0.3%) provisional diagnoses that were in line with the outcome.

When we considered the informative value in relation to higher order internalizing and developmental disorders, we found that just over half of the RLs suggesting anxiety, depression and/or trauma accurately predicted subsequent classifications (Table 4). Indications of autism-related, attention–hyperactivity and/or behavioural problems were predictive in over two-thirds of cases. How well the indications in RLs correlated with later higher order classifications did not differ between girls and boys, different age groups or based on whether there was a previous mental health treatment history (supplementary material).

**Table 4:** Informative value of the referral letter for higher order categories

	Cases/positive RLs	PPV (95% CI)	Non-cases/negative RLs	NPV (95% CI)	Sensitivity	Specificity	LR^+^ (95% CI)	LR^−^ (95% CI)
Anxiety/Depression *n* = 179	121/224	54.0 (49.0–59.0)	58/499	88.4 (86.0–90.4)	67.6 (60.2–74.4)	81.1 (77.5–84.3)	3.57 (2.92–4.37)	0.40 (0.32–0.50)
Anxiety/Depression/ PTSD *n* = 195	137/249	55.0 (50.3–59.6)	58/474	87.8 (85.2–89.9)	70.3 (63.3–76.6)	78.8 (75.1–82.2)	3.31 (2.74–4.00)	0.38 (0.30–0.47)
ASD/ADHD *n* = 391	297/419	70.9 (67.7–73.9)	94/304	69.1 (64.8–73.1)	76.0 (71.4–80.1)	63.3 (57.8–68.5)	2.07 (1.78–2.41)	0.38 (0.31–0.46)
ASD/ADHD/Behavioural disorders *n* = 412	355/505	70.3 (67.7–72.8)	57/218	73.9 (68.5–78.6)	86.2 (82.5–89.4)	51.8 (46.1–57.4)	1.79 (1.58–2.02)	0.27 (0.21–0.35)
All neurodevelopmental/behavioural disorders *n* = 444	383/519	73.8 (71.3–76.2)	61/204	70.1 (64.4–75.2)	86.3 (82.7–89.3)	51.3 (45.2–57.3)	1.77 (1.56–2.01)	0.27 (0.21–0.35)

Depicted are the accuracy metrics in numbers for the combined higher order disorder groups, e.g., Anxiety/Depression depicts the accuracy metrics between RLs containing anxiety and/or depression and the final clinical classification anxiety and/or depression

*PTSD* post-traumatic stress disorder, *ASD* autism spectrum disorders, *ADHD* attention deficit hyperactivity disorder, *PTSD* post-traumatic stress disorder, *ASD* autism spectrum disorders, *ADHD* attention deficit hyperactivity disorder, *PPV* positive predictive value: the number of children with an issued reason for referral who were also classified with that reason for referral as a ratio of the total frequency of that reason for referral, *NPV* negative predictive value: the number of RLs without any indication of the disorder and no final classification, as a ratio of the total number of RLs

Sensitivity = number of children with an issued reason for referral who were also classified with that reason for referral as a ratio of the total prevalence of that diagnostic classification. Specificity = number of RLs without an indication that were also not classified with it as a ratio of the total sample without that diagnostic classification

**Fig. 1:**
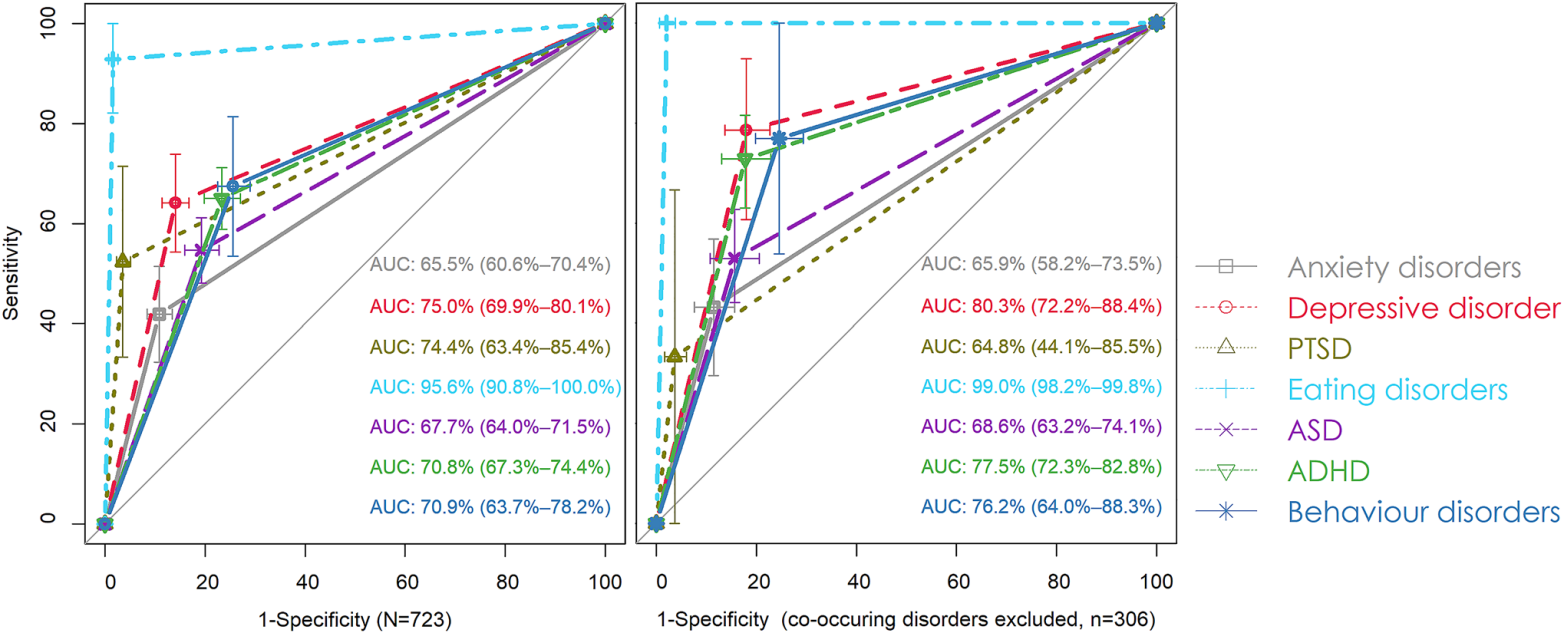
AUROC values of indications made in RLs by disorder group and sample Plotted are the 95% confidence intervals of the sensitivities and specificities, depicted together with the 95% confidence intervals of the AUROC values. The figure on the left presents values of the complete sample (*N* = 723), thus including those with multiple classifications. The figure on the right depicts values in a sample created by excluding cases with co-occurring diagnoses. Note that here the sample size decreased substantially (*n* = 306) as did the number of cases (anxiety disorders *n* = 44, depressive disorder *n* = 28, PTSD *n* = 6, eating disorders *n* = 13, ASD *n* = 102, ADHD *n* = 92, behavioural disorders *n* = 13)

Differences were found with regard to the percentage of specific classifications indicated in RLs (Fig. 1). Youth with anxiety disorders were infrequently referred as such (sensitivity = 41.9%, 95% CI 32.4–51.4), with somewhat higher values for PTSD (52.4%, 95% CI 33.3–71.4) and ASD (54.7%, 95% CI 48.1–61.2). Confidence intervals overlapped for many disorder groups. A notable exception was eating disorders, which were referred with greatest accuracy (sensitivity = 92.9%, specificity = 98.4%). To explore whether the metrics are a result of comorbidity, AUROC values were inspected after removal of those with co-occurring classifications (lower Fig. 1). In absolute terms, sensitivity increased for depressive, eating, and attention-deficit hyperactivity disorders, while at the same time, sample size decreased considerably, limiting the value of these findings.

We then investigated the predictive value of various reasons for referral (Table 5). The highest PPV was found for eating problems, where 67.6% of RLs were concordant with an ensuing eating disorder classification. PPVs varied, with behavioural problems showing the lowest PPV value, followed by trauma, anxiety, depression, autism and attention-hyperactivity problems. The value of the RL in predicting specific disorder groups did not differ between girls and boys, different age groups or depending on treatment history (supplementary material), with the exception of a small age effect for the indication ADHD. Information in the RLs predicted the diagnosis of ADHD better with increasing age (OR = 1.14, 95% CI 1.03–1.27, *p* = 0.026).

**Table 5:** Informative value of RLs for the seven most widespread mental health disorders

Classification	Cases/positive RLs	PPV (95% CI)	Non-cases/negative RLs	NPV (95% CI)	LR^+^ (95% CI)	LR^−^ (95% CI)
Anxiety *n* = 105	44/111	39.6 (32.3–47.5)	551/612	90.0 (88.5–91.4)	3.87 (2.81–5.32)	0.65 (0.55–0.77)
Depressive *n* = 92	59/148	39.9 (34.1–45.9)	542/575	94.3 (92.6–95.6)	4.55 (3.56–5.81)	0.42 (0.32–0.55)
PTSD *n* = 21	11/36	30.6 (20.1–43.5)	677/687	98.5 (97.7–00.1)	14.71 (8.40–25.77)	0.49 (0.32–0.77)
Eating *n* = 27	25/37	67.6 (54.1–78.7)	684/686	99.7 (98.9–99.9)	53.70 (30.34–95.05)	0.08 (0.02–0.29)
ASD *n* = 214	117/215	54.4 (49.0–59.7)	411/508	80.9 (78.4–83.2)	2.84 (2.29–3.52)	0.56 (0.48–0.65)
ADHD *n* = 243	158/270	58.5 (53.9–63.0)	368/453	81.2 (78.4–83.8)	2.79 (2.31–3.36)	0.46 (0.38–0.55)
BD *n* = 43	29/203	14.3 (11.6–17.5)	506/520	97.3 (95.9–98.2)	2.64 (2.06–3.36)	0.44 (0.28–0.67)

*RL* referral letter, *PPV* positive predictive value in percentages, *NPV* negative predictive value in percentages, *LR*^*+*^ positive likelihood ratio, *LR*^*−*^ negative likelihood ratio, *PTSD* post-traumatic stress disorder, *ASD* autism spectrum disorders, *ADHD* attention deficit (hyperactivity) disorders, *BD* behavioural disorders: conduct disorder and oppositional defiant disorder. Frequencies and cross-tabulations are depicted in the supplementary material, as are metrics for the less prevalent disorder groups, the various neurodevelopmental and specific anxiety disorders

Broader investigation of the reasons for referral revealed that a quarter of children referred for mood problems were later classified with an anxiety disorder (24.3%, online supplementary material). The reverse association, i.e., referred for anxiety then classified with depression, was not found. A similar pattern was seen for those eventually diagnosed with behavioural disorders, as they were equally likely to be referred for suggested behavioural problems (14.3%) or trauma (13.9%). Although high raw values were found for some other disorder groups, the frequencies were no more than expected by chance.

Finally, we investigated the informative value of other general problems frequently indicated in RLs (Table 6). Those referred with academic problems were often classified with ADHD (46.4%), and those referred for school attendance problems with an anxiety disorder (42.9%). Half of children referred with possible learning disorders were diagnosed with ADHD. Referral with physical symptoms was significantly associated with a subsequent diagnosis of a depressive disorder (34.4%), and relatively high percentages were also found for anxiety, ASD and ADHD (25%, 25% and 12.5%, respectively). Similarly, around 40% of indications for suicidal ideation or self-harm were subsequently related to a diagnosis of a depressive disorder. Over 80% of children with an indication of bullying or related problems in the social environment were classified with an ASD or ADHD. Other infrequently mentioned problems can be found in the supplementary material.

**Table 6:** Frequency of general reasons for referral per disorder group

	Anxiety disorders	Depressive disorder	PTSD	Eating disorders	ASD	ADHD	Behavioural disorders
Academic problems *n* = 84 st.adj.res	11 (13.1%)	5 (6.0%)	1 (1.2%)	2 (2.4%)	32 (38.1%)	**39 (46.4%)**	7 (8.3%)
	– 0.4	– 2.0	– 1.0	– 0.7	1.8	**2.6**	1.0
School attendance *n* = 28 st.adj.res	**12 (42.9%)**	**8 (28.6%)**	0	0	7 (25.0%)	4 (14.3%)	0
	**4.3**	**2.6**	– 0.9	– 1.1	– 0.5	– 2.2	– 1.4
Learning disorders *n* = 30 st.adj.res	0	0	0	0	8 (26.7%)	**16 (53.3%)**	1 (3.3%) – 0.6
	– 2.3	– 2.1	– 1.0	– 1.1	– 0.4	**2.3**	
Somatic symptoms *n* = 32 st.adj.res	8 (25.0%)	**11 (34.4%)**	0	0	8 (25.0%)	4 (12.5%)	0
	1.7	**3.8**	– 1.0	– 1.1	– 0.6	– 2.6	– 1.5
Problems Sleeping *n* = 18 st.adj.res	4 (22.2%)	4 (22.2%)	2 (11.1%)	1 (5.6%)	2 (11.1%)	7 (38.9%)	0
	0.9	1.2	2.1	0.4	– 1.7	0.5	– 1.1
Suicidal ideation *n* = 53 st.adj.res	10 (18.9%)	**23 (43.4%)**	1 (1.9%)	0	14 (26.4%)	8 (15.1%)	2 (3.8%)
	0.9	**7.0**	– 0.5	– 1.5	– 0.5	– 3.0	– 0.7
Self-harm *n* = 28 st.adj.res	7 (25.0%)	**12 (42.9%)**	**3 (10.7%)**	**3 (10.7%)**	8 (28.6%)	6 (21.4%)	1 (3.6%)
	1.6	**4.9**	**2.5**	**2.0**	– 0.1	– 1.4	– 0.5
Problems with parents *n* = 87	15 (17.2%)	14 (16.1%)	**8 (9.2%)**	3 (3.4%)	16 (18.4%)	25 (28.7%)	**11 (12.6%)**
	0.8	1.0	**3.7**	– 0.2	– 2.4	– 1.3	**2.8**
Bullied/social relatedness *n* = 51	5 (9.8%)	6 (11.8%)	1 (2.0%)	1 (2.0%)	20 (39.2%)	22 (43.1%)	1 (2.0%)
	– 1.0	– 0.2	– 4.0	– 0.7	1.6	1.5	– 1.2

Standardized adjusted residuals depict the discrepancy between observed and expected values and suggest statistical significance at the level of p < 0.05 if >|1.96|

Frequency of the general reasons for referral per disorder group (as a percentage of the total frequency of that reason for referral). A case could be referred for multiple reasons, as well as be classified with multiple diagnoses. Academic problems (ICPC code Z07) were coded when a decline in academic functioning was indicated. Learning disorders (ICPC code P24) were coded when more specific indications were made, such as indications of specific learning disorders, dyslexia, language and speech disorders or developmental coordination disorder. Social relatedness was coded when Loneliness (ICPC code Z04.03) and Relationship problem with friends (Z24) were indicated

## Discussion

The adequate provision of mental healthcare is an ongoing topic and any additional role for RLs beyond an administrative process is a subject of debate within the field. Nonetheless, over half of children in this clinical sample were subsequently classified with at least one condition mentioned in their RL. For higher order combined categories we found PPVs of over 50% for internalizing disorders and over 70% for developmental disorders. Scrutinising PPVs for each of the common diagnostic categories, we found that over two thirds of RLs that suggested eating disorders were in concordance with the outcome. Half of RLs that suggested autism or ADHD as the underlying problem concurred with the later classification. Around two fifths of RLs that mentioned anxiety or depression were later classified as such, and a third of RLs indicating trauma resulted in a classification of PTSD. The least informative reason for referral was behavioural problems, with only one in seven RLs with this indication resulting in the classification of behavioural disorders. Considering sensitivities the highest value was found for eating disorders and the lowest for anxiety disorders. We found no support for an association between predictive value of RLs and estimated length of treatment history, gender, age or level of functional impairment, except for a weak association between higher age and ADHD. Exploration of the reasons for referral more broadly revealed that some general problems such as learning or family problems were often indicated and associated with different outcomes.

Our findings are in line with the broader medical literature, research in various medical specialties suggests that RLs yield some useful information but improvements are necessary. The predictive values we found are similar to the two studies on RLs concerning autism spectrum disorders [14] and non-obsessive–compulsive anxiety disorders [15]. To the best of our knowledge no other studies have been published on the value of RLs in predicting the full range of common diagnostic categories. The differences between disorder groups were, however, mostly parallel to those from general literature on recognition of psychosocial problems. For instance, while RLs are less concordant considering those with anxiety disorders, they were better in including those with depressive disorders [21]. Considering that behavioural problems are mentioned at a fıvefold higher frequency in RLs compared to their prevalence at diagnosis, one might legitimately ask whether referrers label difficult behaviour that may be common in various disorder groups as a behavioural disorder. It is a question for future studies to investigate to what extent it is that referrers pick up or zoom in on these rather externalizing manifestations, or in how far it is a terminological issue and their way to state problems in behaviour.

We also coded and analysed some indications made beyond diagnostic labels. Here we found that problems at school and within the family environment were frequently mentioned. This relates to what is described earlier as the strength and weakness of GPs; ability to adopt a contextual and systemic approach on the one hand [42, 43] and on the other hand focusing less on the internal experience of youth which might impede noticing and recording covert problems, such as anxiety disorders [18]. In the context of the many somatic manifestations of psychosocial problems in youth, a surprising finding was the low prevalence of physical symptoms in RLs. This has been reported earlier in literature on adult mental health [44]. A possible explanation is that once the decision to refer to psychiatry is made, referrers might perceive somatic symptoms as irrelevant [5]. This may also relate to our observation during coding that many RLs seemed to be written as a concise justification of referral [5], rather than a description of the circumstances with the goal of maximum information transfer. Nonetheless, we did not structurally investigate this interesting issue.

About one in ten cases in this referred sample were not classified with a DSM-label and sent back to the referrer or another institution, often primary care. This “wrong referral rate” is up to half the amount suggested in other studies [45] which we relate to the protocol of the institution including pre-intakes by phone. As from the point of view of families and referrers a “back referral” is impactful, we prefer to interpret each registration as a request for help following exhausted resources in general practice [5]. Inspecting RLs within such a perspective could contribute to a mutual understanding of the language and decision-making in both ends of healthcare.

### Strengths and limitations

In a relatively large registration cohort we related information conveyed in RLs to the full breadth of diagnostic outcomes. A strength inherent to the study is that the results present values from everyday practice. A possible concern is the extent to which this single institutional sample reflects the needs of children that register with specialized mental healthcare, as our findings might not be generalizable to centres that operate on another institutional level. Notwithstanding, one could reasonably argue that when investigating the predictive value of RLs it is less the centre’s diversity but the referrers that matters. Since the institution receives referrals from a broad range of referring practices, our results might usefully inform specialized mental health institutions. That said, the current results should be viewed as a first thorough endeavour to the issue of RLs. We choose a priori to examine RLs from general practice only as they are the most frequent referrer and usually the first families turn to for help [46]. Future studies might investigate RLs from other referrers, such as medical specialists and paediatricians to shed light on what differences between referrers exist.

A major strength of our study is the use of the best estimate approach in psychiatry as outcome measure [47]. We included data on patients that were diagnosed using structured assessment as well as clinician judgement following face-to-face interview. The criterion of available structured assessment might have led to a selected sample as those registering in a critical situation are not asked to fill in the DAWBA before the intake interview. Exclusion of these tertiary care patients might either have inflated or deflated agreement. The excluded cases might have had a more complex presentation and thus less concordance between reason of referral and outcome. A part might even not have had a RL as it is not planned care and they arrive through a different route. On the other hand, these youths might have had more marked problems and therewith problems that were better recognizable for the referrer. Nonetheless, a focus on outpatient referrals is preferable in regards generalizability of this first investigation as referrals to specialized healthcare are generally more common and we aimed to gain insight in the value of RLs to child and adolescent mental healthcare.

A downside to extracting clinical data is that clinicians who made the diagnoses had access to RLs, which could potentially inflate agreement between predictor and outcome (“incorporation bias”), although there is insufficient empirical evidence for such effects [48, 49]. In fact, existing literature suggests that most mental health professionals tend to view RLs as incomplete and do not automatically accept information contained in RLs [50]. Moreover, we found PPVs similar to those found in the few available studies. Last but not least, the clinical diagnostic process of the clinic is extensive and elaborate, embracing interviews and questionnaires endorsed by multiple informants and professionals. It is likely that in the presence of this information clinicians will not rely on RLs. That said, replication of current findings in a study setting that ensures complete independence between RLs and diagnoses would lend stronger support to the quantified values.

Another strength of the study was the rigorous coding of information contained in the RLs. We reached good reliability despite multiple labels given to most RLs. In line with the clinical nature of the research question, we aimed to keep the sample as natural as possible, meaning that youth with co-occurring disorders and multiple reasons for referral were included. However, we did not differentiate the main reason for referral or the tentative diagnosis from secondary problems, context, or other symptoms and problems mentioned in RLs. This was impossible given the retrospective design of the study and the differences between RLs in terms of layout and writing style. Basing our coding on first-mentioned issues would have been inadequate, since some GPs first provide extensive background to the referral, others only outline the current situation without providing a clear diagnostic interpretation, whereas many others prefer very short and concise description. Differentiating symptoms and diagnoses presented in RLs might be a topic for future studies as a good RL is proposed to contain an explicit indication of a preliminary diagnosis [43].

A limitation of the study may be the analyses of how the informative value of RLs varies with gender, age, treatment history and level of disfunctioning. Including these four interaction terms in addition to their main effect, together with the imbalance between cases and non-cases, resulted in reduced power. Studies with a larger sample size might differentiate positive and negative agreement between RLs and diagnosis as well, as this might differ depending on these factors. Similarly, our results may have underestimated the informative value of RLs related to the urgency of referral. We differentiated three subsamples of RLs based on the presence of explicit statements of urgency (urgent, serious need, or no explicit statement). Yet we noted descriptions of urgency using more general phrases in RLs that were not included in the two subsamples with explicit statements.

### Implications

The study findings suggest that most RLs do contain valuable information. Nevertheless, an important question is what value is sufficient. On the one hand, none of the diagnostic likelihood ratios we found reached the necessary levels for clinically meaningful use, with the exception of indications for eating disorders. On the other hand, as we might cautiously infer from the moderate AUROCs found in this study, RLs may be almost as valuable as some structured assessment instruments in discriminating psychiatric classifications [16, 51, 52]. However, this assertion should be placed in perspective of the numbers and the context of referrals. As the study considered prevalent disorders and a selected sample, even the high values we found imply a major cost of false omissions when absolute numbers are considered [21]. From the perspective of referrers [53], attributing subsequently divergent diagnoses as inaccurate would lack the necessary nuance. Specialized healthcare populations are epitomised by inherently complex problems and the need for elaborate diagnosis is a valid reason for referral. Putting aside expectations of high accuracy, our results support use of RLs as a node of information in the diagnostic work-up. Beyond their effect on diagnosis and allocation, incorporating RLs in the assessment process may have a welcome side-effect as it might potentially ameliorate families perception of fragmented care [45].

In countries, where the GP has a gatekeeper role, content guidelines and formats are defined and embedded in health records to help improve RLs. Accordingly, the RL is an integral component of a GPs’ training and continuing medical education [43, 54–56]. The sensitivity and specificity values found in this study might help inform curricula.

Another finding with clinical implications concerns the ICPC codes included in RLs. When GPs register a code they also write out a short description, often in just two or three words. We observed that these descriptions often suggested a disorder or symptom that diverged from the ICPC code they had registered and copied to the RL. This suggests that the ICPC codes communicated in a RL have limited significance in specialized mental health services, and in research using automatized analysis in medical records. Finally, guidelines on coding could be improved as the study revealed some limits of the P and Z chapters of the ICPC coding system. There are some inconsistencies between symptom and disorder codes, as some codes for important problems are lacking, whereas multiple codes exist for some less prevalent symptoms. In recent years a sub-code for autism spectrum disorders has been added, for example, and most GPs in our sample seemed to use it as intended.

## Conclusion

In this study, we investigated the symptoms and provisional diagnoses described in RLs to child and adolescent mental healthcare. We conclude that, contrary to widespread clinical anecdotes, RLs appear to hold informative value and might add to the clinical process in child and adolescent psychiatry. Future studies of RLs may shed light on other important dimensions of utility and quality. Among these are the clarity and completeness of the information conveyed, the investigation and treatment requested, and how these factors relate to the diagnostic work-up and treatment families eventually receive [13, 57, 58]. Another essential question relates to the factors explaining individual differences between RLs. Quantification of the complete process between referral and assessment is necessary to stimulate a mutual understanding of strengths and weaknesses—at both the referring and receiving end in healthcare—and thus help inform the day-to-day diagnostic process.

## Supplementary Information

Below is the link to the electronic supplementary material.

Supplementary file1 (DOCX 29 kb)

Supplementary file2 (DOCX 54 kb)

## Data Availability

No additional data are available for this study in repositories. Inquiries concerning the data may be made to the corresponding author.

## Declarations

### Reporting guidelines

Please find the STARD checklist in the online supplementary.
