# Supplementary material for: Informative value of referral letters from general practice for child and adolescent mental healthcare
Source: Eur Child Adolesc Psychiatry. 2021 Aug 21;32(2):303–15. doi: 10.1007/s00787-021-01859-7 (PMC9970945; doi:10.1007/s00787-021-01859-7)
Supplement: Supplementary file 1 — Supplementary file1 (DOCX 29 kb) [file 787_2021_1859_MOESM1_ESM.docx]

# Online supplementary material

Aydin, Crone, Siebelink, Numans, Vermeiren, & Westenberg. 2021. Informative value of referral letters from general practice for child and adolescent mental healthcare. European child and adolescent psychiatry.

| Supplementary Table 1  Origin of the referral letters N=1259 | |
| --- | --- |
|  | **n (%)** |
| General Practitioner (GP) | 689 (54.7) |
| GP and another referrer | 34 (2.7) |
| Specialists |  |
| Psychiatry | 61 (4.8) |
| Pediatrics | 172 (13.7) |
| Rehabilitation doctors | 13 (1.0) |
| Others | 30 (2.4) |
| Local youth teams |  |
| Youth and family centres | 217 (17.2) |
| Municipal Health services | 48 (3.8) |
| Juvenile probation officer | 29 (2.3) |

In the Netherlands, a formal referral to child and adolescent psychiatry proceeds either via general practice, specialized health centers (hospitals), the local youth welfare offices, or via youth protection boards. A total of 723 (57.4%) RLs were from general practice, and 34 of these cases had a RL from a GP and another referrer. For these cases we extracted only information from the RL originating from general practice.

| Supplementary Table 2  Chance corrected agreement | |
| --- | --- |
|  | κ (95% CI) |
| Anxiety disorders | .81 (.73 - .86) |
| Depressive disorder | .82 (.71 - .94) |
| PTSD | .77 (.57 - .96) |
| Eating disorders | .87(.69 - 1.00) |
| ASD | .90 (.82 - .98) |
| ADHD | .90 (.83 - .97) |
| Behavioral | .77 (.63 - .66) |

Chance corrected agreement, Kappa values, per disorder group computed over a random selection of 150 RLs that were coded by the author who coded all RLs and the three second coders who each coded a set of 50 letters.

| Supplementary Table 4  How often and which ICPC-codes were written in referral letters? | | | | | |
| --- | --- | --- | --- | --- | --- |
|  | First | Second | Third | Fourth | Fifth |
| A Unspecified | 23 (3.2) | 20 (2.8) | 9 (1.2) | 6 (0.8) | 4 (0.6) |
| B Blood and Immune mechanism | 1 (0.1) | 1 (0.1) | - | - | 1 (0.1) |
| D Digestive | 9 (1.2) | 7 (1.0) | 7 (1) | - | 2 (0.3) |
| F Eye | 6 (0.8) | 3 (0.4) | 2 (0.3% | 3 (0.4) | - |
| H Ear | 5 (0.7) | 3 (0.4) | 2 (0.3) | 4 (0.6) | 1 (0.1) |
| K Cardiovascular | 4 (0.5) | 5 (0.7) | 1 (0.1) | 1 (0.1% | - |
| L Musculoskeletal | 8 (1.1) | 13 (1.8) | 8 (1.1) | 1 (0.1) | 4 (0.6) |
| N Neurological | 10 (1.4) | 5 (0.7) | 5 (0.7) | 3 (0.4) | - |
| P Psychological | 176 (24.3) | 65 (9.0) | 29 (4.0) | 11 (1.5) | 5 (0.7) |
| R Respiratory | 34 (4.7) | 29 (4.0) | 20 (2.8) | 13 (1.8) | 2 (0.3) |
| S Skin | 20 (2.8) | 21 (2.9) | 11 (1.5) | 5 (0.7) | 5 (0.7) |
| T Endocrine/ metabolic | 16 (2.2) | 9 (1.2) | 3 (0.4) | 1 (0.1) | 1 (0.1) |
| U/W Urological/ Pregnancy | 2 (0.2) | 2 (0.3) | 7 (1.0) | - | 1 (0.1) |
| X/Y Female/ Male genital | - | 5 (0.7) | 2 (0.3) | 2 (0.3) | 1 (0.1) |
| Z Social problems | 17 (2.4) | 6 (0.8 ) | 2 (0.3) | 1 (0.1) | 2 (0.3) |
| No ICPC-code written in RL | 392 (54.2) | 529 (73.2) | 617 (85.3) | 672 (92.9) | 695 (96.1) |

Depicted are the number (%) of ICPC-codes written in RLs. Frequently issued specific codes by the referrer were A12-Allergy (n=18), R96-Asthma (n=46), and S87-Eczema (n=37)[1]. Frequencies are not depicted on the level of the specific codes as a result of low frequencies and the differences we observed between these registered ICPC-codes and their accompanying short textual description (also written in RLs). Latter suggests probable unreliable use of the ICPC-code at that level (as discussed in the discussion section in the main manuscript).

| Supplementary Table 5  Odds of classification per disorder group | | | | |
| --- | --- | --- | --- | --- |
|  |  | | OR | 95% CI |
| Anxiety disorders | | RL | 5.93 | 3.73 – 9.43 |
|  |  | RL+ | 4.76 | 2.90 – 7.83 |
|  |  | Age | 1.15 | 1.07 – 1.24 |
|  |  | Gender | 1.23 | 0.76 – 1.97 |
|  |  | History | 1.28 | 0.72 – 2.26 |
|  |  | CGAS | 1.00 | 0.97 – 1.04 |
| Depression | | RL | 10.89 | 6.73 – 17.62 |
|  |  | RL+ | 4.79 | 2.83 – 8.10 |
|  |  | Age | 1.42 | 1.26– 1.59 |
|  |  | Gender | 1.70 | 0.98 – 2.96 |
|  |  | History | 1.07 | 0.56 – 2.03 |
|  |  | CGAS | 0.98 | 0.95 – 1.02 |
| PTSD | | RL | 29.79 | 11.58 – 76.63 |
|  |  | RL+ | 45.47 | 15.31 – 135.06 |
|  |  | Age | 1.10 | 0.95 – 1.28 |
|  |  | Gender | 2.63 | 0.89 –7.78 |
|  |  | History | 2.41 | 0.49 – 11.73 |
|  |  | CGAS | 0.93 | 0.86 – 1.00 |
| Eating disorders | | RL | 808. 36 | 170.43 – 3834.19 |
|  |  | RL+ | 681.78 | 95.95 – 4844.36 |
|  |  | Age | 1.55 | 0.97 – 2.50 |
|  |  | Gender | 3.10 | 0.26 – 36.67 |
|  |  | History | 1.10 | 0.18 – 6.84 |
|  |  | CGAS | 0.90 | 0.81 – 1.01 |
| Autism spectrum disorders | | RL | 5.06 | 3.57 – 7.16 |
|  |  | RL+ | 5.17 | 3.48– 7.68 |
|  |  | Age | 0.94 | 0.89 – 0.99 |
|  |  | Gender | 0.47 | 0.31 – 0.71 |
|  |  | History | 1.46 | 0.94 – 2.25 |
|  |  | CGAS | 0.93 | 0.91 – 0.96 |
| ADHD | | RL | 6.11 | 4.36 – 8.56 |
|  |  | RL+ | 7.39 | 5.09 – 10.74 |
|  |  | Age | 0.91 | 0.86 – 0.96 |
|  |  | Gender | 0.59 | 0.40 – 0.88 |
|  |  | History | 0.89 | 0.58 – 1.35 |
|  |  | CGAS | 1.00 | 0.97 – 1.03 |
| Behavioral disorders | | RL | 6.02 | 3.11 – 11.66 |
|  |  | RL+ | 7.14 | 3.45 – 14.77 |
|  |  | Age | 1.04 | 0.94 – 1.16 |
|  |  | Gender | 1.08 | 0.54 – 2.17 |
|  |  | History | 1.86 | 0.74 – 4.68 |
|  |  | CGAS | 0.95 | 0.90 – 0.99 |

Values depict the odds ratios (OR) resulting from logistic regression analyses with disorder group as outcome. PTSD= Post traumatic stress disorder. ADHD= attention deficit hyperactivity disorders. Each upper row (RL) depicts the univariate analysis with only referral letter as predictor. Second to fifth rows (RL+) depict odds corrected for the main effects of age, gender, treatment history, and CGAS score. Age and CGAS were included as continuous variables. The reference for gender are boys. Psychiatric treatment history is included with ‘0 no treatment history’ being the reference. In a third block the interaction terms RL*age, RL*gender, RL*history and RL*CGAS were added to test for the possibility that some disorder groups are mentioned more often in RLs depending on these factors. No significant interaction effects were found, except for an indication of ADHD*age (OR=1.14, 95% CI 1.03-1.27, *p*=.026).

| Supplementary Table 6  Accuracy metrics for the most prevalent psychiatric disorder groups | | | | | | | |  |  |  |  |
| --- | --- | --- | --- | --- | --- | --- | --- | --- | --- | --- | --- |
|  |  | Anxiety disorders  n=105 | Mood disorders  n=92 | PTSD  n=21 | Eating disorders  n=27 | ASD  n=214 | ADHD  n=243 | Behavioral Disorders  n=43 | Personality disorder  n=34 | Psychosomatic disorders  n=17 | Re. attachm. & Disin. s. eng. d.  n=16 |
| Anxiety ICPC  n=111 | TP (Se, PPV) | **44 (41.9, 39.6)** | 17 (18.5, 15.3) | 3 (14.3, 2.7) | 3 (11.1, 2.7) | 28 (13.1, 25.2) | 28 (11.5, 25.2) | 5 (11.6, 4.5) | 7 (20.6, 6.3) | 4 (23.5, 3.6) | 3 (18.8, 2.7) |
|  | st.adj.res. | **8.2** | 0.9 | -0.1 | -0.6 | -1.1 | -2.0 | -0.7 | 0.9 | 0.9 | 0.4 |
|  | TN (Sp) | **551 (89.2)** | 537 (85.1) | 594 (84.6) | 588 (84.5) | 426 (83.7) | 397 (82.7) | 574 (84.4) | 585 (84.9) | 599 (84.8) | 599 (84.7) |
| Mood ICPC  n=148 | TP (Se, PPV) | **36 (34.3, 24.3)** | **59 (64.1, 39.9)** | 6 (28.6, 4.1) | 7 (25.9, 4.7) | 35 (16.4, 23.6) | 30 (12.3, 20.3) | 5 (11.6, 3.4) | **15 (44.1, 10.1)** | 7 (41.2, 4.7) | 2 (12.5, 1.4) |
|  | st.adj.res. | **3.8** | **11.1** | 0.9 | 0.7 | -1.8 | -3.9 | -1.5 | **3.5** | 2.1 | -0.8 |
|  | TN (Sp) | **506 (81.9)** | **542 (85.9)** | 560 (79.8) | 555 (79.7) | 396 (77.8) | 362 (75.4) | 537 (79.0) | **556 (80.7)** | 565 (80.0) | 561 (79.3) |
| Trauma ICPC  n=36 | TP (Se, PPV) | 7 (6.7, 19.4) | 2 (2.2, 5.6) | **11 (52.4, 30.6)** | 1 (3.7, 2.8) | 3 (1.4, 8.3) | 8 (3.3, 22.2) | 5 (11.6, 13.9) | 3 (8.8, 8.3) | 1 (5.9, 2.8) | 1 (6.3, 2.8) |
|  | st.adj.res. | 0.9 | -1.3 | **10.1** | -0.3 | -2.9 | -1.5 | 2.1 | 1.1 | 0.2 | 0.2 |
|  | TN (Sp) | 589 (95.3) | 597 (94.6) | **677 (96.4)** | 661 (95.0) | 476 (93.5) | 452 (94.2) | 649 (95.4) | 656 (95.2) | 671 (95.0) | 672 (95.0) |
| Eating ICPC  n=37 | TP (Se, PPV) | 7 (6.7, 18.9) | 6 (6.5, 16.2) | 1 (4.8, 2.7) | **25 (92.6, 67.6)** | 4 (1.9, 10.8) | 1 (0.4, 2.7) | 1 (2.3, 2.7) | 4 (11.8, 10.8) | 0 (-) | 0 (-) |
|  | st.adj.res. | 0.8 | 0.7 | -0.1 | **21.0** | -2.6 | -4.1 | -0.9 | 1.8 | -1.0 | -0.9 |
|  | TN (Sp) | 589 (95.3) | 601 (95.2) | 667 (95.0) | **684 (98.3)** | 476 (93.5) | 444 (92.5) | 644 (94.7) | 656 (95.2) | 669 (94.8) | 670 (94.8) |
| Autism ICPC  n=215 | TP (Se, PPV) | 18 (17.1, 8.4) | 14 (15.2, 6.5) | 1 (4.8, 0.5) | 1 (3.7, 0.5) | **117 (54.7, 54.4)** | 68 (28.0, 31.6) | 9 (20.9, 4.2) | 4 (11.8, 1.9) | 1 (5.9, 0.5) | 4 (25.0, 1.9) |
|  | st.adj.res. | -3.1 | -3.3 | -2.5 | -3.0 | **9.5** | -0.7 | -1.3 | -2.3 | -2.2 | -0.4 |
|  | TN (Sp) | 421 (68.1) | 430 (68.1) | 488 (69.5) | 482 (69.3) | **411 (80.7)** | 333 (69.4) | 474 (69.7) | 478 (69.4) | 492 (69.7) | 496 (70.2) |
| ADHD ICPC n=270 | TP (Se, PPV) | 20 (19.0, 7.4) | 16 (17.4, 5.9) | 6 (28.6, 2.2) | 6 (22.2, 2.2) | 70 (32.7, 25.9) | **158 (65.0, 58.5)** | 11 (25.6, 4.1) | 4 (11.8, 1.5) | 1 (5.9, 0.4) | 5 (31.3, 1.9) |
|  | st.adj.res. | -4.2 | -4.2 | -0.8 | -1.7 | -1.7 | **10.9** | -1.6 | -3.2 | -2.7 | -0.5 |
|  | TN (Sp) | 368 (59.5) | 377 (59.7) | 438 (62.4) | 432 (62.1) | 309 (60.7) | **368 (76.7)** | 421 ( 61.9) | 423 (61.4) | 437 (61.9) | 442 (62.5) |
| Behavior-al ICPC  n=203 | TP (Se, PPV) | 20 (19.0, 9.9) | 11 (12.0, 5.4) | 5 (23.8, 2.5) | 1 (3.7, 0.5) | 75 (35.0, 36.9) | 64 (26.3, 31.5) | **29 (67.4, 14.3)** | 3 (8.8, 1.5) | 1 (5.9, 0.5) | **10 (62.5, 4.9)** |
|  | st.adj.res. | -2.2 | -3.7 | -0.4 | -2.9 | 2.7 | -0.7 | **5.9** | -2.6 | -2.1 | **3.1** |
|  | TN (Sp) | 435 (70.4) | 439 (69.6) | 504 (71.8) | 494 (71.0) | 381 (74.9) | 341 (71.0) | **506 (74.4)** | 489 (71.0) | 504 (71.4) | **514 (72.7)** |
| Persona-lity ICPC  n=52 | TP (Se, PPV) | 9 (8.6, 17.3) | **15 (16.3, 28.8)** | **5 (23.8, 9.6)** | 1 (3.7, 1.9) | 12 (5.6, 23.1) | 16 (6.6, 30.8) | 6 (14.0, 11.5) | **8 (23.5, 15.4)** | 1 (5.9, 1.9) | **9 (56.3, 17.3)** |
|  | st.adj.res. | 0.6 | **3.6** | **3.0** | -0.7 | -1.1 | -0.5 | 1.8 | **3.8** | -0.2 | **7.7** |
|  | TN (Sp) | 575 (93.0) | **594 (94.1)** | **655 (93.3)** | 645 (92.7) | 469 (92.1) | 444 (92.5) | 634 (93,2) | **645 (93.6)** | 655 (92.8) | **664 (93.9)** |
| Somati-sation ICPC n=32 | TP (Se, PPV) | 8 (7.6, 25.0) | **11 (12.0, 34.4)** | 0 (-) | 0 (-) | 8 (3.7, 25.0) | 4 (1.6, 12.5) | 0 (-) | 1 (2.9, 3.1) | **5 (29.4, 15.6)** | 0 (-) |
|  | st.adj.res. | 1.7 | **3.8** | -1.0 | -1.1 | -0.6 | -2.6 | -1.5 | -0.4 | **5.1** | 0.9 |
|  | TN (Sp) | 594 (96.1) | **610 (96.7)** | 670 (95.4) | 664 (95.4) | 485 (95.3) | 452 (94.2) | 648 (95.3) | 658 (95.5) | **679 (96.2)** | 675 (95.5) |

Columns: frequency of diagnoses as classified in specialized mental healthcare. Rows: frequency of tentative diagnoses in RLs. TP= true positives, number of youth that had an indication of the diagnosis they are classified with in their RLs. Se= sensitivity, ratio TPs to the frequency of the diagnoses, in percentages. PPV= positive predictive value, ratio TPs to the frequency of diagnoses in RLs. St.adj.res.= standardized adjusted residual, computed as a measure of co-occurrence beyond chance. Depicts the discrepancy between observed and expected values and suggest statistical significance at the level of p<.05 when it exceeds |1.96|. Given the many comparisons made, we set a higher boundary of |3.0| [2]. TN= true negatives, number of youth without the classification and no indication in the RL. Sp= specificity, ratio TN to the whole sample without the disorder group, in percentages. ASD=autism spectrum disorders.

ADHD= attention deficit hyperactivity. Re. attachm. & Disin. s. eng. d.= reactive attachment & disinhibited social engagement disorder.

Results suggest that a quarter of children referred for mood problems were later classified with an anxiety disorder (24.3%, 36/105). The reverse association, i.e. referred with anxiety then classified with depression, was not foud. Nor was it accounted for by co-occurrence of depression in those classified with an anxiety disorder: after exclusion of the comorbid cases (n=16) the number of children with anxiety disorders that were referred for depressive disorders remained similar (28.1%, 25/89). A similar pattern was seen for those evenntually diagnosed with behavioural disorders, as they were equally likely to be referred for suggested behavioural problems (14.3%), and or trauma (13.9%, 5/43). Again this association remained after exclusion of the cases with comorbid PTSD (16.0%, 4/25). Although high raw values were found for some other disorder groups, the frequencies were no more than what could be expected by chance.

Assuming that the reason of consult for Reactive attachment disorder and Disinhibited social engagement disorder might differ from those with PTSD, we included only PTSD from the Trauma and stressor-related disorders chapter in the tabulation with indications of trauma made in RLs. Adjustment disorders were not cross-tabulated as a result of their small sample size and since they can not be the only diagnosis in, or reason of referral to, specialized mental healthcare according to the Dutch legislation. For their relative severe nature, personality disorders were mentioned relative frequently in the RLs, but had a low sensitivity (23.5%). A suggestion of personality or attachment problems in RLs was significantly related to a classification of depression (28.8%) and PTSD (9.6%). This association decreased with about a third, to respectively 20.5% (9/44, st.adj.res.=2.1.) and 6.8% (3/44, st.adj.res.=2.0), when those with a co-occuring personality disorders were excluded. An indication of somatisation disorder in RLs was to some extent related to a later classification of somatisation disorder (PPV=15.6%). When next to somatisation disorder (which has a specific ICPC-code: P75), other bodily symptoms were also counted as an indication of somatic symptom and related disorders, both the sensitivity and PPV increased, to respectively 58.8% (10/17), and 25% (10/40). This (combining P75-somatisation disorder and indications of other bodily symptoms and physical complaints) did not change associations with other disorder groups.

| Supplementary Table 7  Cross tabulation of reasons of referral and clinical status for  the low prevalence disorder groups that were not included in the main manuscript | | |
| --- | --- | --- |
|  | **PPV of the disorder specific labels** | **Sensitivity when all labels from the chapter are combined** |
| Intellectual disability | 1/8 (12.5) | 16/21 (76.2) |
| Communication disorder | 1/12 (8.3) | 17/18 (94.4) |
| Motor disorders | 5/ 14 (35.7) | 11/14 (78.6) |
| Specific learning disorder | 11/30 (36.7) | 28/38 (73.7) |
| + Other Neurodevelopmental Disorders + ASD +  ADHD | 322/435 (74.0) |  |
| + high IQ | 323/439 (73.6) |  |
| Separation anxiety disorder | - | 4/8 (50.0) |
| Specific phobia | 0/2 (0.0) | 2/6 (33.30 |
| Social anxiety disorder | 1/7 (14.3) | 10/16 (62.5) |
| Panic disorder | 1/5 (20.0) | 5/8 (62.5) |
| Agoraphobia | - | 1/1 (100.0) |
| Generalized anxiety disorder | 0/2 (0.0) | 24/47 (51.1) |
| Anxiety disorder not otherwise specified | - | 6/28 (21.4) |
| OCD | 5/15 (33.3) | 5/8 (62.5) |

Separation anxiety disorder, agoraphobia, and anxiety disorder NOS do not have specific ICPC-codes. Hence no disorder specific PPVs’ are depicted in these rows. Sensitivity is depicted on the level of the neurodevelopmental disorders chapter, the anxiety disorders chapter and for obsessive compulsive disorders (including trichotillomania n=2). Extant yet infrequent reasons of referral were emotion dysregulation (n=21), self-image (19) and game addiction (4) [3].

| **Supplementary Table 8**  **Extended table reasons of referral per disorder groups** | | | | | | | |
| --- | --- | --- | --- | --- | --- | --- | --- |
|  | **Anxiety disorders**  **n=105** | **Mood disorders**  **n=92** | **PTSD**  **n=21** | **Eating disorders**  **n=27** | **ASD**  **n=214** | **ADHD**  **n=243** | **Behavioral Disorders**  **n=43** |
| **Study problems**  n=84 St. adj. res. | 11 (13.1%)  -0.4 | 5 (6.0%)  -2.0 | 1 (1.2%)  -1.0 | 2 (2.4%)  -0.7 | 32 (38.1%)  1.8 | 39 (46.4%)  2.6 | 7 (8.3%)  1.0 |
| **School attendance problems**  n=28 St. adj. res. | 12 (42.9%)  4.3 | 8 (28.6%)  2.6 | 0  -0.9 | 0  -1.1 | 7 (25.0%)  -0.5 | 4 (14.3%)  -2.2 | 0  -1.4 |
| **Perfectionism/ fear of failure**  n=20 St. adj. res. | 6 (30.0%) | 3 (15.0%) | 1 (5.0%) | 1 (5.0%) | 6 (30.0%) | 4 (20.0%) | 1 (5.0%) |
|  | 2.0 | 0.3 | 0.6 | 0.3 | 0.0 | -1.3 | -0.2 |
| **High IQ**  n=16 St. adj. res. | 4 (25.0%) | 2 (12.5%) | 0 | 0 | 4 (25.0%) | 4 (25.0%) | 2 (12.5%) |
|  | 1.2 | 0.0 | 0.7 | -0.8 | -0.4 | -0.7 | 1.1 |
| **Intellectual disabilities**  n=8 St. adj. res. | 0 | 1 (12.5%) | 1(12.5%) | 0 | 3 (37.5%) | 2 (25.0%) | 0 |
|  | -1.2 | 0.0 | 1.6 | -0.6 | 0.5 | -0.5 | -0.7 |
| **Learning disorders**  n=30 St. adj. res. | 0 | 0 | 0 | 0 | 8 (26.7%) | 16 (53.3%) | 1 (3.3%)  -0.6 |
|  | -2.3 | -2.1 | -1.0 | -1.1 | -0.4 | 2.3 |  |
| **Communication problems**  n=12 St. adj. res. | 1 (8.3%) | 0 (0.0% ) | 0 | 0 | 5 (41.7%) | 5 (41.7%) | 0 |
|  | -6 | -1.3 | -0.6 | -0.7 | 0.9 | 0.6 | -0.9 |
| **Somatic symptoms**  n=32 St. adj. res. | 8 (25.0%) | 11 (34.4%) | 0 | 0 | 8 (25.0%) | 4 (12.5%) | 0 |
|  | 1.7 | 3.8 | -1.0 | -1.1 | -0.6 | -2.6 | -1.5 |
| **Headaches**  n=7 | 0 | 2 (28.6%) | 0 | 0 | 0 | 0 | 0 |
|  | -1.1 | 1.3 | -0.5 | -0.5 | -1.7 | -1.9 | -0.7 |
| **Pain-Fatigue**  n=17 | 6 (35.3%) | 6 (35.3%) | 0 | 0 | 6 (35.3%) | 3 (17.6%) | 0 |
|  | 2.5 | 2.8 | -0.7 | -0.8 | 0.5 | -1.4 | -1.0 |
| **Stomach/ Bowel** | 2 (22.2%) | 3 (33.3%) | 0 | 0 | 2 (22.2%) | 0 | 0 |
| n=9 | 0.7 | 1.9 | -0.5 | -0.6 | -0.5 | -2.1 | -0.8 |
| **Fainting/ Powerlessness**  n=3 | 2 (66.7%) | 1 (33.3%) | 1 (33.3%) | 1 (33.3%) | 0 | 0 | 0 |
|  | 2.6 | 1.1 | 3.1 | 2.7 | -1.1 | -1.2 | -0.4 |
| **Hyperventilation** | 1 (50.0%) | 0 | 0 | 0 | 0 | 1 (50.0%) | 0 |
| n=2 | 1.4 | -0.5 | -0.2 | -0.3 | -0.9 | 0.5 | -0.4 |
| **Problems Sleeping**  n=18 St. adj. res. | 4 (22.2%) | 4 (22.2%) | 2 (11.1%) | 1 (5.6%) | 2 (11.1%) | 7 (38.9%) | 0 |
|  | 0.9 | 1.2 | 2.1 | 0.4 | -1.7 | 0.5 | -1.1 |
| **Suicidal ideation**  n=53 St. adj. res. | 10 (18.9%) | 23 (43.4%) | 1 (1.9%) | 0 | 14 (26.4%) | 8 (15.1%) | 2 (3.8%) |
|  | 0.9 | 7.0 | -0.5 | -1.5 | -0.5 | -3.0 | -0.7 |
| **Self Harm**  n=28 St. adj. res. | 7 (25.0%) | 12 (42.9%) | 3 (10.7%) | 3 (10.7%) | 8 (28.6%) | 6 (21.4%) | 1 (3.6%) |
|  | 1.6 | 4.9 | 2.5 | 2.0 | -0.1 | -1.4 | -0.5 |
| **(Sexual) Violence**  n=8 St. adj. res. | 1 (12.5%) | 1 (12.5%) | 5 (62.5%) | 1 (12.5%) | 0 | 1 (12.5%) | 0 |
|  | -2.0 | 0 | 10.1 | 1.3 | -1.8 | -1.3 | -0.7 |
| **Problems with parents**  n=87 St. adj. res. | 15 (17.2%) | 14 (16.1%) | 8 (9.2%) | 3 (3.4%) | 16 (18.4%) | 25 (28.7%) | 11 (12.6%) |
|  | 0.8 | 1.0 | 3.7 | -0.2 | -2.4 | -1.3 | 2.8 |
| **Bullied-Social relatedness**  n=51 St. adj. res. | 5 (9.8%)  -1.0 | 6 (11.8%)  -0.2 | 1 (2.0%)  -4.0 | 1 (2.0%)  -0.7 | 20 (39.2%)  1.6 | 22 (43.1%)  1.5 | 1 (2.0%)  -1.2 |

Frequency (%) of the reasons of referral per disorder group, i.e. referred with the reason of referral in the row and classified with the disorder group in de column. Below each row percentage are standardized adjusted residual values depicted. A case could be referred for multiple reasons as well as be classified with multiple disorders.

**References**

1. Hartveit, M., et al., *Recommended content of referral letters from general practitioners to specialised mental health care: a qualitative multi-perspective study.* BMC Health Serv Res, 2013. **13**: p. 329.

2. Haberman, S.J., *Adjusted st. res. the Analysis of Frequency Data,*. 1974, Chicago: University of Chicago Press.

3. Scottish Association for Mental Health and Information Services Division Scotland and NIHS, *Rejected Referrals Child and Adolescent Mental Health Services (CAMHS)*, in *A qualitative and quantitative audit*, T.S. Group Scotland, Edinburgh EH6 5NA PPDAS433246 (06/18), Editor. 2018, The Scottish Government.
